# Tobacco Smoke

**Published:** 1885-03

**Authors:** 


### TOBACCO SMOKE.

M. Zulinsky has recently published, says the *British Medical Journal*, in a Polish Medical paper, the results of a large series of experiments on men and animals, made for the purpose of ascertaining the physiological action of tobacco smoke on animals. He has found that the smoke is a powerful poison, even in very small quantities. In the case of a man, tobacco smoke, when not inhaled too freely, is deleterious only to a certain extent. M. Zulinski declares that the poisonous character of the smoke is not entirely due to the nicotine which it contains. Tobacco smoke rendered free from nicotine remains poisonous, though not to so great a degree as before. The second poisonous principle is alklaoid collodine.

Carbonic oxide, hydrocyanic acid, and other noxious principles are also contained in tobacco smoke. The bad effects of excessive smoking depend very much both on the kind of tobacco consumed and on the manner of consuming it. In cigar smoking the greatest amount of poison is inhaled, in cigarettes much less, in pipes still less, while those who indulge in the nargileh, or any similar luxury where the smoke is drawn through water take tobacco in its least mischievious form. Such are M. Zulinsky's conclusions. There can be little doubt that many of the light-colored tobaccos have been partially bleached in order to give them that pale tint which moderate smokers believe to be an infallible indication of mildness. The decolorising agent is suspected to be in many places a deleterious chemical compound. Some of the light tobaccos smoke exceedingly hot, owing to the quantity of woody fibre which they contain. This is especially the case with "birds-eye," which is cut near the stalk of the leaf, the slices of the mid-rib, thick in this part of the leaf, giving this variety of tobacco the characteristic appearance whence it derives it name. "Birds-eye" is apt to cause slight inflammation of the tongue, on account of the irritant character and heat of its smoke, and together with the other light tobaccos, must act very prejudicially in elderly smokers, who may be prone to cancer of the tongue or lip. Dark tobaccos are readily adulterated; but, when pure, they are probably the most healthy for pipe-smoking.

---

Proof of Death.—If most people are afraid of anything, it is being buried alive. That cases do happen where it is very difficult even for the experienced physician to determine whether a person is really, or but apparently dead, without his having recourse to means which, while they would at once settle the dispute, would place life, if it really still existed, in jeopardy, may be judged from the fact that the French Academy, some ten or fifteen years ago, offered a prize of forty thousand francs for the discovery of some means by which even the inexperienced may determine whether in a given case death had ensued or not. A physician obtained the prize. He had discovered the following well-known phenomenon: If the hand of the suspected person is held towards the candle or other artificial light, with the fingers stretched and one touching the other, and one looks through the spaces between the fingers and the light, there appears a scarlet red color where the faces touch each other, due to the still circulating fluid blood, as it shows itself through the transparent, not yet congested tissues; but when life is extinct this phenomenon at once ceases. The most extensive and thorough trials established the truth of this observation, and the prize was awarded to its discoverer.

What to do—If a man faint, place him flat on his back, loosen his clothing and let him alone.

